# Electrospinning CaCO_3_/Porous PLA Nanofibers for Daytime Radiative Cooling

**DOI:** 10.3390/polym18131580

**Published:** 2026-06-25

**Authors:** Yangyang Sun, Changnai Yang, Mengge Li, Xiaomin Zeng, Dengkun Su, Shiyi Pan, Yu Zhang, Qiong Jiang, Shizhe Lin

**Affiliations:** 1 Guangxi Key Laboratory of Optical and Electronic Materials and Devices, College of Materials Science and Engineering, Guilin University of Technology, Guilin 541004, China; 2 Medical College of Guangxi University, Guangxi University, Nanning 530004, China

**Keywords:** porous structure, interface, nanofiber, passive daytime radiative cooling

## Abstract

To develop high-performance and eco-friendly passive daytime radiative cooling (PDRC) materials, calcium carbonate (CaCO_3_)/porous polylactic acid (PLA) nanofibers were fabricated via electrospinning. This fabrication utilized PLA as the matrix and 40 nm CaCO_3_ nanoparticles as fillers, with ambient humidity controlled above 85%RH during electrospinning. The resulting nanofibers possessed numerous CaCO_3_/PLA interfaces and porous surface structures. Experimental results demonstrated that the CaCO_3_/porous PLA nanofibers achieved a solar reflectivity of ~92.3%, significantly exceeding that of PLA (~72.1%), CaCO_3_/PLA (~86.0%), and porous PLA (~79.6%) nanofibers. During outdoor testing, CaCO_3_/porous PLA nanofibers exhibited optimal PDRC performance with a temperature reduction of ~10.3 °C, representing a 6.1 °C improvement compared to PLA nanofibers. This enhancement is attributed to synergistic light-scattering sites generated by surface porosity and CaCO_3_/PLA interfaces, which collectively strengthen solar spectrum scattering. Furthermore, significant morphological degradation was observed after 80-day soil burial, confirming biodegradability. This study proposes a facile strategy for developing high-performance eco-friendly PDRC materials.

## 1. Introduction

Passive daytime radiative cooling (PDRC) has been identified as a promising strategy for achieving sub-ambient cooling without energy consumption. Specifically, incident solar radiation in the 0.3~2.5 μm wavelength range is reflected by these cooling materials to minimize heat gain, while thermal radiation is emitted through the atmospheric transparency window in the 8~13 μm range toward the cold universe at ~3 K [1,2]. Owing to this dual functionality, PDRC is rendered highly attractive for applications including building energy efficiency, outdoor personal thermal management, vehicle cooling, and photovoltaic panel thermal regulation [3,4]. In contrast to traditional cooling methods (such as compression refrigeration), which are associated with considerable electricity consumption and greenhouse gas emissions, PDRC is regarded as a passive and environmentally sustainable alternative [5]. However, a stringent requirement for ultrahigh solar reflectivity is imposed by the intense daytime solar irradiance, which typically reaches ~1000 W m^−2^ under clear skies [6,7,8]. If solar reflectivity is insufficient, the radiative heat loss will be exceeded by the absorbed solar energy, thereby nullifying the cooling effect. Therefore, the enhancement of solar reflectivity has been recognized as a central challenge in the development of high-performance PDRC materials.

Recent studies have focused on the design of PDRC materials exhibiting high solar reflectivity [9,10]. Early investigations were concentrated on multilayer photonic structures, by which the reflection and emission spectra could be precisely controlled [11]. Excellent spectral selectivity was demonstrated by such structures. However, their large-scale applications have been constrained by complex fabrication processes and high cost [12]. Electrospinning is a versatile and scalable technique for fabricating nanofibers with controllable morphology, high porosity, and large specific surface area [13,14,15]. The fiber networks produced by electrospinning extend photon transport pathways and promote multiple scattering interfaces, which are highly desirable for achieving high solar reflectivity [16,17,18,19]. Moreover, electrospinning nanofibers can be engineered with hierarchical structures, including surface pores and bead-on-string morphologies, to further enhance light scattering [20,21]. Additionally, the high porosity facilitates infrared emission by providing a large surface area for thermal radiation [14,22]. However, these high-performance electrospinning PDRC materials are predominantly derived from non-biodegradable polymers such as polyvinylidene fluoride, polyimide, or fluorinated polyurethane [19,23,24,25,26]. The widespread use of such materials not only accelerates the depletion of non-renewable resources but also causes persistent environmental pollution due to their poor degradability. Consequently, an urgent need exists to develop electrospun nanofibers that combine high solar reflectivity with environmental friendliness and biodegradability.

Polylactic acid (PLA) is a biodegradable polyester derived from renewable feedstocks such as corn starch, sugarcane, or cassava [23,27]. PLA exhibits outstanding processability, mechanical flexibility, and eco-compatibility, thereby rendering it an ideal matrix [28]. Furthermore, the intrinsic absorption of PLA in the atmospheric transparency window (8~13 μm) is advantageous for thermal emission, which can be attributed to the vibrational modes of carbonyl, carbon–oxygen, and carbon–hydrogen bonds [27,29,30]. PLA-based PDRC materials have been investigated in several studies, which have encompassed fabric structures. For example, PLA nonwoven fabrics have been investigated for personal thermal management and promising cooling effects [31]. However, limited solar scattering capability is exhibited by conventional PLA nanofibers owing to the relatively low refractive index and the absence of internal scattering features [32]. Therefore, the enhancement of the solar reflectivity of PLA nanofibers has been identified as a promising avenue for research.

To overcome this limitation, high-refractive-index inorganic fillers are widely incorporated into the PLA matrix, whereby heterogeneous scattering interfaces are created [19]. When such interfaces are encountered by light, strong scattering is caused by the abrupt change in refractive index [33]. The scattering efficiency is proportional to the square of the refractive index contrast between the filler and the matrix. Among various fillers, calcium carbonate (CaCO_3_) is distinguished by its natural abundance and low cost [34]. More importantly, a refractive index of ~1.60 is exhibited by calcite CaCO_3_, which is substantially higher than that of PLA at ~1.45, and efficient light scattering at CaCO_3_/PLA interfaces is consequently enabled [35]. In addition, CaCO_3_ has been widely used in polymer composites for packaging and biomedical applications due to its biodegradability and non-toxicity [25,36]. More importantly, favorable mid-infrared emissivity is also exhibited by CaCO_3_ due to its light absorption bands [37]. It has been confirmed by these advantages that calcium carbonate constitutes a promising eco-friendly filler for PDRC. Beyond filler incorporation, light scattering can be further enhanced by tailoring the surface morphology of nanofibers. The surface structure of nanofibers is critically influenced by the ambient humidity during electrospinning [38]. Under high-humidity conditions, water vapor is condensed on the jet surface, phase separation is induced, and a porous surface morphology is formed after solvent evaporation [39]. This humidity-induced phase separation technique has been successfully applied to various polymers to create porous nanofibers for filtration, tissue engineering, and catalysis. Abundant air–polymer interfaces are introduced by the porous structures, and these act as additional scattering sites for incident light [40].

Herein, CaCO_3_/porous PLA nanofibers were fabricated via electrospinning under high humidity (>85%RH), with PLA, CaCO_3_/PLA, and porous PLA nanofibers prepared for comparison. Humidity-induced phase separation generated porous surface structures, embedding CaCO_3_ nanoparticles (NPs, ~40 nm) within the PLA matrix to establish CaCO_3_/PLA interfaces. The resulting nanofibers exhibited a high specific surface area of ~38.49 m^2^ g^−1^ and a pore volume of ~0.1207 cm^3^ g^−1^, which are the highest among the four nanofibers. The Young’s modulus and tensile strength were measured as ~63.1 MPa and ~1.66 MPa, respectively, indicating favorable mechanical properties. Moreover, the cooling performance remained stable after 70 cycles of simulated solar irradiation and after 48 h of immersion in water or saline solution, demonstrating good environmental stability. Comprehensive investigation of microstructural morphology, solar reflectivity, and indoor/outdoor cooling performance indicated that the porous surfaces and CaCO_3_/PLA interfaces synergistically enhanced PDRC performance. Furthermore, CaCO_3_/porous PLA nanofibers exhibit flexibility, breathability, and biodegradability. This study proposes a facile strategy for fabricating high-performance eco-friendly PDRC materials.

## 2. Materials and Methods

### 2.1. Materials

PLA (4032D, M_w_ = 20 × 10^4^) was purchased from NatureWorks Co., Ltd. (Minnetonka, MN, USA). Dichloromethane (DCM; purity ≥ 99.9%) and *N*,*N*-Dimethylformamide (DMF) were purchased from Shanghai Titan Technology Co., Ltd. (Shanghai, China). CaCO_3_ NPs (~40 nm) were obtained from Beijing Boyu Hi-Tech New Materials (Beijing, China).

### 2.2. Preparation of Various Nanofibers

1.5 g of PLA was dissolved in a mixed solvent containing 11.5 g DCM and 2.5 g DMF under magnetic stirring at room temperature to yield a PLA solution. Subsequently, ~40 nm CaCO_3_ NPs were introduced into the PLA solution at a mass ratio of 6:4 (PLA:CaCO_3_), followed by additional agitation to ensure homogeneous dispersion, thus producing the CaCO_3_/PLA solution. Electrospinning was conducted using a 19 G needle with an applied voltage of +15 kV, a feed rate of 0.09 mL h^−1^, and a collection distance of 15 cm. Nanofibers were fabricated with either smooth or porous surfaces by modulating ambient humidity during processing. Specifically, smooth PLA and CaCO_3_/PLA nanofibers were prepared under 45 ± 5%RH, whereas porous PLA and CaCO_3_/porous PLA nanofibers were obtained at >85%RH. In this process, a handmade electrospinning system was used, consisting of a high-voltage power supply (Tianjin Dongwen High-Voltage Power Supply Co., Ltd., Tianjin, China, DW-N303-1ACH2), a syringe pump (Jiaozuo Yanhang Power Co., Ltd., Jiaozuo, China, QHZS-001), and a drum collector (Qingdao Pansi Technology Co., Ltd., Qingdao, China; rotation speed 0–1350 rpm).

### 2.3. Characterization Methods

#### 2.3.1. Morphology and Structural Characterization of the Nanofibers

The surface morphology of nanofibers was characterized by field-emission scanning electron microscopy (FE-SEM; Tokyo, Japan, Hitachi S-4800). Fiber diameter distribution was statistically analyzed using ImageJ software (version 1.54p). The crystal structure was characterized by X-ray diffraction (XRD; PANalytical, Almelo, The Netherlands, X’pert PRO) with Cu Kα radiation (λ = 0.15418 nm) over a scanning range of 5–80°.

#### 2.3.2. Optical Performance Testing of the Nanofibers

Solar reflectivity (0.3–2.5 μm) was measured via ultraviolet–visible–near-infrared spectrophotometry (UV–vis–NIR, Shimadzu UV-3600i Plus, Kyoto, Japan) equipped with an integrating sphere, using a BaSO_4_ whiteboard as the reflectance reference. Atmospheric window emissivity (8–13 μm) was determined by Fourier-transform infrared spectroscopy (FTIR, Thermo Nicolet iS50, Madison, WI, USA) coupled with a gold-coated integrating sphere, while sample temperature was maintained at 25 °C.

#### 2.3.3. Flexibility, Permeability and Degradability Testing of the Nanofibers

Flexibility was qualitatively evaluated by wrapping the nanofibers around a glass rod (~0.8 cm diameter) and observing whether fracture occurred.

To evaluate permeability, water vapor transmission was assessed using the gravimetric method at 38 °C. The water vapor transmission rate (WVTR) was calculated as follows [41,42]:

(1) $$\begin{array}{c} WVTR\;=\;\frac{\Delta m}{A\cdot t}\; \end{array}$$

where Δ*m* denotes mass loss during testing, *A* represents the effective transmission area, and *t* indicates test duration.

Degradability was investigated through soil burial experiments. CaCO_3_/porous PLA nanofibers (3 cm × 3 cm) were buried in open containers filled with natural soil. Containers were placed outdoors under natural conditions to simulate realistic degradation. Samples were retrieved every 16 days and visually inspected for macroscopic changes.

#### 2.3.4. Radiative Cooling Performance Testing of the Nanofibers

Outdoor tests were conducted under clear skies in Guilin, China (25°17′14″ N, 110°19′31″ E) using a handmade apparatus (external dimensions 27.0 cm × 20.5 cm × 20.5 cm, thickness 2 cm). Sample temperature, ambient humidity, and solar irradiance were monitored in real-time via multichannel thermocouples (Suzhou Te’ansi Electronic Industry Co., Ltd., Suzhou, China, TA612C), hygrometers, and photometric sensors (TES-1333R, Taipei, Taiwan, China). Indoor simulations were performed by placing samples in a controlled environment (~60 °C), with surface temperature distribution recorded by infrared thermography (TESTO 865, Lenzkirch, Germany). Radiant cooling power was computed based on the thermal balance equation. Nighttime tests followed identical procedures under zero-solar-irradiation conditions.

### 2.4. Statistical Analysis

Fiber diameter distributions were determined by measuring at least 30 nanofibers using ImageJ software, and subsequent statistical analysis was performed. Cooling performance and mechanical properties, including tensile strength and Young’s modulus, were assessed in at least five independent replicates. Data are expressed as mean ± standard deviation, with error bars representing the standard deviation.

## 3. Results and Discussion

### 3.1. Fabrication and Characterization of the CaCO_3_/Porous PLA Nanofibers

**Figure 1:**
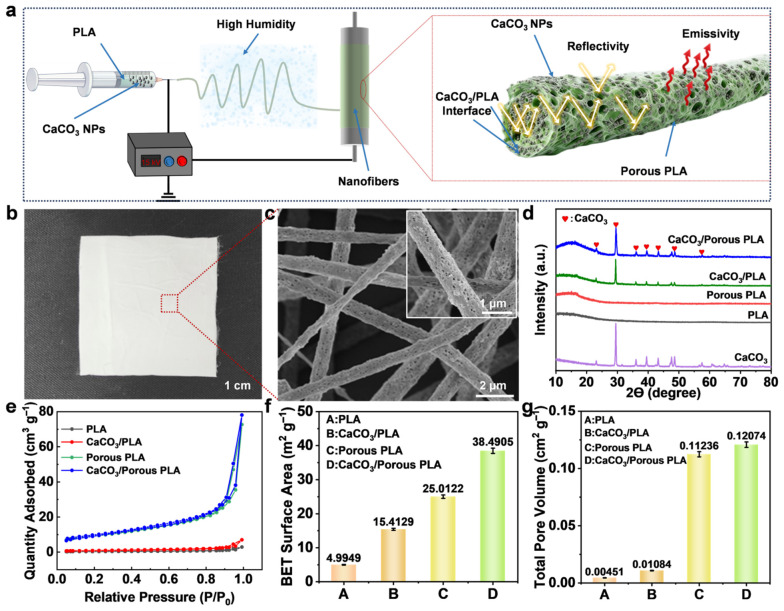
(**a**) Fabrication schematic, (**b**) photograph and (**c**) SEM images of the CaCO_3_/porous PLA nanofibers. (**d**) XRD patterns of the CaCO_3_ and four nanofibers. (**e**) Nitrogen adsorption–desorption isotherms, (**f**) surface area and (**g**) pore volume of various samples.

As illustrated in Figure 1a, CaCO_3_/porous PLA nanofibers were fabricated via electrospinning with humidity-induced phase separation. Notably, the introduction of CaCO_3_ NPs into the PLA matrix generated heterogeneous interfaces within the composite nanofibers. Simultaneously, phase separation was induced during solidification by controlling the spinning ambient humidity, resulting in abundant surface porosity. The nanofibers possessed the CaCO_3_/porous PLA interface and the porous structure, which collectively enhanced solar light scattering. Additionally, the porous surface architecture provided increased sites for infrared radiation emission [43,44,45]. As shown in Figure 1b, the CaCO_3_/porous PLA nanofibers exhibited a uniform white appearance. As demonstrated in Figure 1c and Figure S1, CaCO_3_/porous PLA nanofibers with diameters of ~1 μm were interconnected into a non-woven network, exhibiting extensive surface porosity. For comparison, PLA, CaCO_3_/PLA, and porous PLA nanofibers were prepared under identical processing parameters but with varied spinning humidity or solution compositions (Figures S2 and S3). XRD patterns of the four nanofibers and CaCO_3_ NPs are shown in Figure 1d. Both PLA and porous PLA nanofibers exhibited broad amorphous halos, indicating low crystallinity. Conversely, distinct CaCO_3_ diffraction peaks were observed in CaCO_3_/PLA and CaCO_3_/porous PLA composites, confirming successful incorporation of CaCO_3_ without crystal structure degradation [46,47]. EDS mapping of CaCO_3_/porous PLA nanofibers has also been performed. As shown in Figure S4, the mapping reveals uniform distributions of C, O, and Ca throughout the nanofiber, with no noticeable aggregation of the CaCO_3_ nanoparticles. Furthermore, high-resolution SEM images overlaid with EDS maps demonstrate that numerous CaCO_3_ nanoparticles are embedded within the PLA matrix, generating abundant CaCO_3_/PLA interfaces. Thermogravimetric analysis (TGA) further characterized the thermal decomposition (Figure S5).

The nitrogen adsorption–desorption curves of the four nanofibers are shown in Figure 1e. For all nanofibers, a gradual increase in the adsorption branch was observed at P/P_0_ < 0.1, which was attributed to monolayer-to-multilayer nitrogen adsorption on the material surface. As P/P_0_ increased, a divergence between the adsorption and desorption branches emerged, and hysteresis loops were developed, which indicated the presence of porous structures. At P/P_0_ > 0.95, a sharp rise in the adsorbed amount was detected, a phenomenon typically attributed to capillary condensation within macropores or voids. Notably, the highest adsorption capacity was sustained by the CaCO_3_/porous PLA nanofibers across the entire P/P_0_ range, confirming that these nanofibers possessed the most abundant porous architecture. The specific surface areas of the four nanofibers are shown in Figure 1f. The PLA nanofibers exhibited a specific surface area of ~4.99 m^2^ g^−1^. Upon the incorporation of CaCO_3_ NPs, the specific surface area of the nanofibers was raised to ~15.41 m^2^ g^−1^, which indicated that additional adsorption sites were provided by the CaCO_3_ NPs dispersed within the PLA matrix. When the porous structure was fabricated, the specific surface area of the porous PLA nanofibers was elevated to ~25.01 m^2^ g^−1^. Remarkably, the highest specific surface area (38.49 m^2^ g^−1^), was attained by the CaCO_3_/porous PLA nanofibers, which revealed a pronounced synergistic effect between CaCO_3_ NPs and surface pores. Similarly, the pore volumes are shown in Figure 1g. The pore volume of the PLA nanofibers was measured to be ~0.0045 cm^3^ g^−1^. For the CaCO_3_/PLA nanofibers, the pore volume was increased to ~0.0108 cm^3^ g^−1^, a value slightly higher than that of the PLA, which may be attributed to a small quantity of pores formed between NPs or at the NP/fiber interfaces. In contrast, the pore volume of the porous PLA nanofibers was substantially increased to ~0.1124 cm^3^ g^−1^, demonstrating that the construction of the porous structure significantly expanded the space of internal pores. Furthermore, the CaCO_3_/porous PLA nanofibers reached the maximum pore volume of ~0.1207 cm^3^ g^−1^, confirming the synergistic effect exerted by the co-presence of CaCO_3_ NPs and the porous structure. The specific surface area and pore volume data collectively displayed a consistent ascending order, with the values increasing in the PLA, CaCO_3_/PLA, porous PLA, and CaCO_3_/porous PLA. This trend reveals that the pore characteristics of the nanofibers were effectively enhanced by either the introduction of CaCO_3_ NPs or the fabrication of porous structures, whereas the best pore structure optimization was realized through their synergistic integration.

### 3.2. Mechanical and Permeability Properties of the Nanofibers

**Figure 2:**
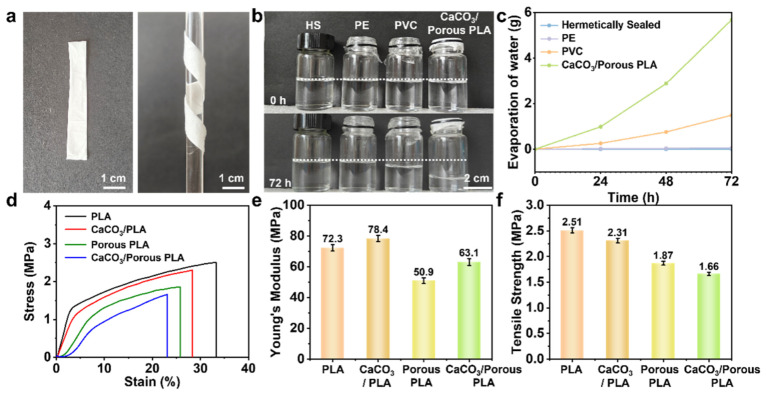
(**a**) Flexibility and (**b**) breathability test of the CaCO_3_/porous PLA nanofibers. (**c**) WVTR curves of various samples. (**d**) Stress–strain curves, (**e**) Young’s modulus and (**f**) tensile strength of the four nanofiber samples.

Figure 2a demonstrates the mechanical flexibility of CaCO_3_/porous PLA nanofibers, which tightly adhere to a glass rod without cracking. Figure 2b compares water evaporation in sealed bottles using four materials: a hermetically sealed (HS) bottle, polyethylene (PE) film, polyvinyl chloride (PVC) film, and CaCO_3_/porous PLA nanofibers. After 72 h, negligible water loss was observed in the airtight bottle. In contrast, the PE-sealed bottle exhibited slight water reduction (0.057/10 g/g), while the PVC-sealed bottle showed a more substantial decrease (1.491/10 g/g). Notably, the CaCO_3_/porous PLA nanofibers achieved the highest mass loss of 5.682/10 g/g (Figure 2c). Based on these measurements, the WVTR of the CaCO_3_/porous PLA nanofibers was calculated as ~251.2 g m^−2^ h^−1^. These results confirm the excellent water vapor permeability of the fabricated CaCO_3_/porous PLA nanofibers. Furthermore, the WVTR of PLA, porous PLA, and CaCO_3_/PLA nanofibers was measured (Figure S6), which proved highly useful for understanding the structural influence on moisture permeability. The results indicate that the PLA nanofibers exhibited the lowest WVTR (~70.0 g m^−2^ h^−1^), followed by CaCO_3_/PLA nanofibers (~127.1 g m^−2^ h^−1^) and porous PLA nanofibers (~199.7 g m^−2^ h^−1^). Consistent with expectations, the CaCO_3_/porous PLA achieved superior performance (~251.2 g m^−2^ h^−1^). This marked enhancement can be attributed to the fact that the higher pore volume (Figure 1g) facilitated water vapor permeation.

The stress–strain curves of the four nanofibers are shown in Figure 2d. The highest tensile strength was recorded for the PLA nanofibers. With the fabrication of the porous structure, a reduction in tensile strength was observed. Similarly, a decrease in tensile strength was induced by the incorporation of CaCO_3_ NPs. The Young’s modulus values are summarized in Figure 2e. A value of ~72.3 MPa was determined for the PLA nanofibers. An increase to ~78.4 MPa was recorded for the CaCO_3_/PLA nanofibers, whereas the porous PLA nanofibers exhibited a decrease to ~50.9 MPa. The CaCO_3_/porous PLA nanofibers were measured at ~63.1 MPa. The tensile strength values are shown in Figure 2f. The PLA nanofibers exhibited a tensile strength of ~2.51 MPa, which was reduced to ~2.31 MPa for the CaCO_3_/PLA nanofibers, further decreased to ~1.87 MPa for the porous PLA nanofibers, and lowered to ~1.66 MPa for the CaCO_3_/porous PLA nanofibers. Among all nanofibers, the highest Young’s modulus (~78.4 MPa) and the second-highest tensile strength (~2.31 MPa) were displayed by the CaCO_3_/PLA nanofibers, whereas lower values for both properties were recorded for the porous PLA nanofibers (~50.9 MPa and ~1.87 MPa, respectively). This discrepancy was attributed to the distinct types of structural defects in the two systems. In the CaCO_3_/PLA nanofibers, the CaCO_3_ NPs acted as stress concentration sites, which contributed to load bearing and restricted polymer chain mobility, thereby endowing the nanofibers with a relatively high Young’s modulus. In contrast, the effective load-bearing area was reduced by the pores in the porous PLA nanofibers, and severe stress concentrations were introduced at the pore boundaries, causing pronounced decreases in both tensile strength and Young’s modulus. The aforementioned analysis further elucidated why the CaCO_3_/porous PLA nanofibers exhibited a higher Young’s modulus but a lower tensile strength compared with the porous PLA nanofibers. Although the tensile properties of the CaCO_3_/porous PLA nanofibers were not the most prominent among the four nanofibers, the tensile strength (~1.66 MPa) and Young’s modulus (~63.1 MPa) were maintained at sufficiently high levels to preserve adequate structural integrity for applications.

### 3.3. Optical Properties, Thermal Imaging and Cycling Stability of the Nanofibers

**Figure 3:**
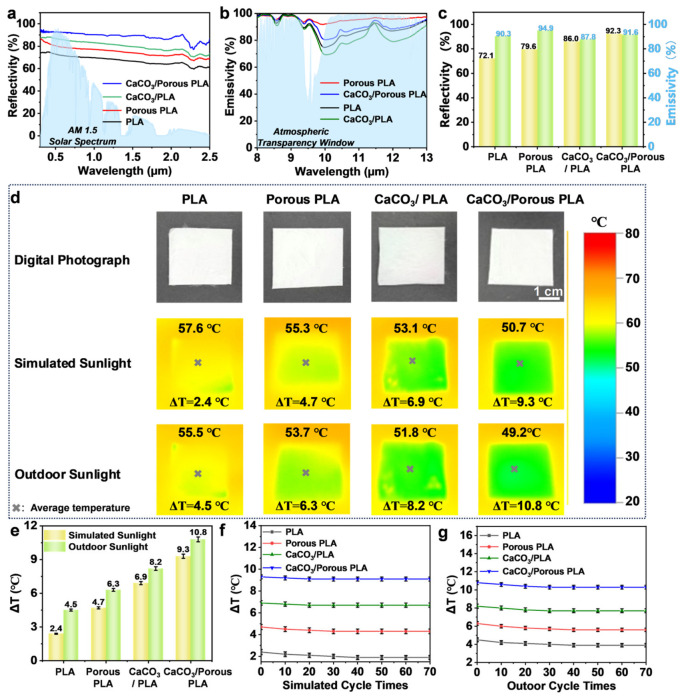
(**a**) Solar reflectivity, (**b**) atmospheric window emissivity spectra and (**c**) a summary of average reflectivity and emissivity of the four nanofibers. (**d**) Infrared thermal images under indoor simulated daylight and outdoor sunlight. (**e**) Corresponding temperature reduction bar chart. (**f**) Temperature reduction variation over 70 on–off cycles under simulated sunlight and (**g**) outdoor sunlight.

The solar reflectivity and atmospheric transparency window emissivity constitute fundamental optical parameters for evaluating radiative cooling performance. High solar reflectivity minimizes solar radiation absorption, thereby reducing external heat gain. In contrast, high emissivity facilitates efficient thermal dissipation toward the cold universe through the atmospheric transparency window. Figure 3a,c present solar reflectivity spectra of the four nanofibers over the 0.3–2.5 μm wavelength range. The PLA nanofibers exhibited the lowest reflectivity, averaging ~72.1% across the solar spectrum. Due to abundant air/PLA interfaces introduced by surface pores [48,49,50], the porous PLA nanofibers achieved an increased reflectivity of ~79.5%. Incorporation of CaCO_3_ NPs within the PLA matrix enhanced light scattering via heterogeneous interfaces [33,51,52], resulting in a reflectivity of ~86.0% for CaCO_3_/PLA nanofibers. Conversely, the CaCO_3_/porous PLA nanofibers demonstrated the highest reflectivity in the solar spectrum (~92.3%), attributed to synergistic effects of surface porosity and interfaces. Figure 3b,c display emissivity spectra within the atmospheric transparency window (8–13 μm). The porous PLA nanofibers showed the highest emissivity (~94.9%), while those of PLA, CaCO_3_/PLA, and CaCO_3_/porous PLA were ~90.3%, ~87.8%, and ~91.6%, respectively. This phenomenon confirms that porous structures provide additional emission sites, thereby enhancing infrared emittance [53]. Notably, despite a marginally lower emissivity compared to porous PLA nanofibers, CaCO_3_/porous PLA maintained high performance (~91.6%).

The superior optical properties (92.3% reflectivity and 91.6% emissivity) of the CaCO_3_/porous PLA nanofibers were primarily attributed to three structural features. First, abundant pores on nanofiber surfaces generated numerous air/PLA interfaces, enhancing scattering of incident solar radiation. Second, uniformly dispersed CaCO_3_ NPs established high-density CaCO_3_/PLA interfaces, further promoting light scattering. Third, the nonwoven fibrous network prolonged the optical path, enabling repeated scattering among pores, interfaces, and nanofiber interstices. Consequently, these synergistic effects endowed CaCO_3_/porous PLA nanofibers with exceptional solar reflectivity. Simultaneously, the intrinsic mid-infrared vibrational absorption of PLA and CaCO_3_, and abundant radiation sites from the porous structure sustained high atmospheric transparency window emissivity. Ultimately, integration of porous architectures and heterogeneous interfaces enabled outstanding optical performance for PDRC applications. Under simulated solar irradiation indoors, the nanofibers were positioned on the testing platform. Figure 3d,e demonstrate that the CaCO_3_/porous PLA nanofibers exhibited the lowest surface temperature (~50.7 °C) with a corresponding temperature reduction of ~9.3 °C. Consistently, outdoor thermal imaging under natural sunlight demonstrated an identical trend (a lowest surface temperature of ~49.2 °C and a maximum temperature reduction of ~10.8 °C). These results indicate that the CaCO_3_/porous PLA nanofibers possess optimal cooling performance in high-temperature environments. This observation aligns closely with its highest solar reflectivity (Figure 3a,c), confirming that enhanced solar reflection capability is pivotal for improving cooling efficiency. The temperature difference variation in the CaCO_3_/porous PLA nanofibers under repeated indoor simulated solar illumination is shown in Figure 3f and Figure S7. After 70 cycles, the temperature difference was slightly reduced from ~9.3 °C to ~9.1 °C, corresponding to a decrease of 0.2 °C. This result indicates that the material possesses favorable cyclic stability under indoor simulated illumination conditions. The temperature difference variation under repeated outdoor sunlight is shown in Figure 3g. Similarly, after 70 cycles, the temperature difference was slightly decreased from ~10.8 °C to ~10.3 °C, corresponding to a reduction of 0.5 °C. The above results confirm that no significant attenuation of the PDRC performance was observed for the CaCO_3_/porous PLA nanofibers upon repeated use.

### 3.4. Outdoor Radiative Cooling Properties of the CaCO_3_/Porous PLA Nanofibers

**Figure 4:**
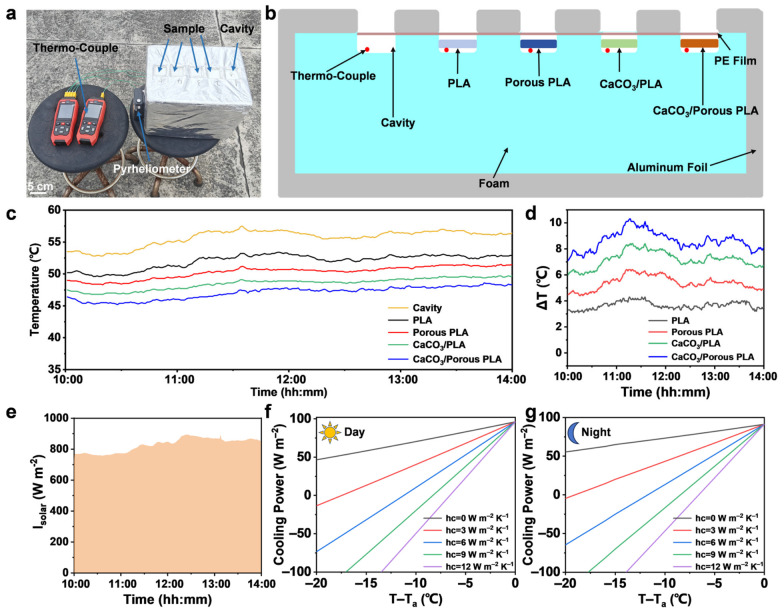
(**a**) Photograph and (**b**) cross-sectional schematic of the outdoor test setup. (**c**) Daytime outdoor cooling performance (temperature 25–32 °C, humidity 31–67%RH, wind speed 5–19 km h^−1^, Guilin, China) and (**d**) temperature reduction during outdoor testing of various nanofibers. (**e**) Solar intensity curve. (**f**) Daytime and (**g**) nighttime cooling power of the CaCO_3_/porous PLA nanofibers.

Figure 4a,b illustrate photographs and a cross-sectional schematic of the outdoor testing device, respectively. The apparatus was constructed with foam as the primary insulating layer, while its inner walls were coated with aluminum foil to minimize external thermal interference. Samples were mounted atop the chamber and encapsulated with polyethylene (PE) film. Temperatures were monitored in real-time via thermocouples to evaluate the cooling performance of different samples under actual outdoor conditions. Figure 4c shows temperature curves of four nanofibers during outdoor testing. The CaCO_3_/porous PLA nanofibers consistently exhibited the lowest temperature throughout the test period, indicating superior outdoor cooling performance. Conversely, the PLA nanofibers recorded the highest temperature, reflecting the weakest solar reflectivity. Both porous PLA and CaCO_3_/PLA nanofibers demonstrated intermediate temperatures. Figure 4d illustrates the temperature differentials between nanofibers and the cavity. The largest absolute differential (~10.3 °C) was observed for CaCO_3_/porous PLA, significantly surpassing those of PLA (~4.2 °C), porous PLA (~6.4 °C), and CaCO_3_/PLA (~8.4 °C). These findings demonstrate that either porous structures or CaCO_3_ NPs individually enhance cooling performance, whereas their synergistic combination yields the most significant improvement. Figure 4e shows the recorded solar irradiance profile during testing, with a peak value of ~890 W m^−2^. Based on the thermal equilibrium equation (detailed calculations are provided in the Supplementary Information), the daytime radiative cooling power of CaCO_3_/porous PLA nanofibers was calculated as ~96.1 W m^−2^ (Figure 4f), while the nighttime radiative cooling power reached ~91.2 W m^−2^ (Figure 4g). These results confirm the capability of nanofibers to achieve effective temperature reduction under both solar radiation and nighttime conditions. Furthermore, nighttime radiative cooling tests were conducted (Figure S8), which further validate its sustained radiative heat dissipation capacity. As detailed in Table S1, solar reflectance, emissivity, cooling power, and mechanical properties are systematically encompassed.

### 3.5. Environmental Stability and Degradability of the CaCO_3_/Porous PLA Nanofibers

**Figure 5:**
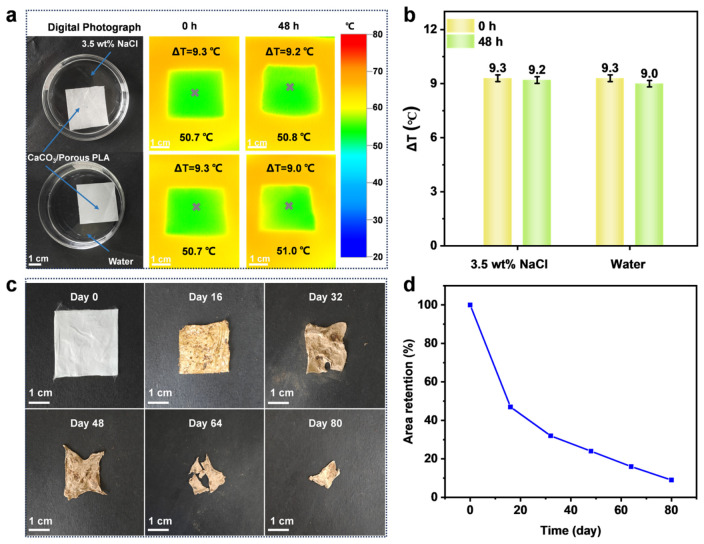
(**a**) Photographs and IR thermal images before/after immersion, ✕: Average Temperature, (**b**) corresponding temperature reduction, (**c**) photographs during soil burial at different times, and (**d**) normalized residual area versus burial time.

Figure 5a demonstrates optical and infrared thermal images of the CaCO_3_/porous PLA nanofibers after immersion in water and a 3.5 wt% sodium chloride (NaCl) solution for 0 and 48 h. No obvious dissolution, fragmentation, or particle detachment was observed in either medium, indicating favorable tolerance of the material in aqueous environments. The infrared thermal images visually revealed the surface temperature distribution of the nanofibers before and after immersion. The corresponding temperature reductions are shown in Figure 5b. Before immersion, a temperature reduction of ~9.3 °C was recorded for the nanofibers. After immersion in water for 48 h, the temperature reduction decreased to ~9.0 °C, corresponding to a decline of 0.3 °C. Following immersion in the NaCl solution for 48 h, the temperature reduction was slightly lowered to ~9.2 °C, corresponding to a decrease of 0.1 °C. These results indicate that only a marginal attenuation of the cooling performance occurred after treatment with aqueous and saline solutions, demonstrating that the nanofibers can maintain effective PDRC functionality in humid and salt-spray environments.

Figure 5c shows photographs of CaCO_3_/porous PLA nanofibers sampled at distinct intervals during soil burial at 0, 16, 32, 48, 64, and 80 d. Initially (0 d), the nanofibers exhibited intact and homogeneous surfaces. Subsequently, progressive surface damage and pore formation were observed with prolonged burial duration. By 48 d, wrinkling and fragmentation initiated at the nanofiber edges. Ultimately, only minor fragments persisted at 80 d, indicating substantial structural disintegration. These results demonstrate the notable degradability of the CaCO_3_/porous PLA nanofibers, attributed to the degradation of PLA within soil environments. The area variation in the CaCO_3_/porous PLA nanofibers during soil burial was quantified in Figure 5d, with the initial area (0 d) normalized to 100%. After 16 d of burial, the remaining area was reduced to 47%, which further decreased to 32% after 32 d, 24% after 48 d, 16% after 64 d, and only 9% after 80 d. This progressive reduction in area confirmed a gradual biodegradation process. As shown in Figure S9, after soil was removed from the surfaces as thoroughly as possible, the sample mass steadily decreased from 0.027 g on Day 16 to 0.005 g on Day 80, with the residual mass at Day 80 being only 18.5% of that recorded at Day 16. This trend was consistent with the morphological observations in Figure 5c, thereby validating the environmentally benign characteristics of the CaCO_3_/porous PLA nanofibers.

## 4. Conclusions

In summary, CaCO_3_/porous PLA nanofibers were fabricated via electrospinning utilizing humidity-induced phase separation. Comprehensive characterization including SEM, XRD, UV–Vis–NIR spectroscopy, infrared thermal imaging, and outdoor daytime radiative cooling testing was systematically conducted to investigate the microstructural morphology, crystalline structure, solar reflectivity, and cooling performance. Experimental results demonstrate that the CaCO_3_/porous PLA nanofibers exhibited optimal properties. Under infrared thermal imaging, temperature differentials of ~9.3 °C (indoor) and ~10.8 °C (outdoor) were observed relative to ambient conditions. Furthermore, a maximum temperature reduction of ~10.3 °C was achieved during outdoor testing, corresponding to a cooling power of ~96.1 W m^−2^. The cooling performance remained stable after 70 cycles of simulated solar irradiation and after 48 h of immersion in water or saline solution, demonstrating good environmental stability. This enhanced performance is predominantly attributed to synergistic effects between the surface porous architecture and CaCO_3_/PLA interfaces. Additionally, the nanofibers demonstrated notably high WVTR (~251.2 g m^−2^ h^−1^) and favorable degradability (significant structural disintegration after 80 days of soil burial). Collectively, this strategy provides a facile and scalable approach for developing high-performance eco-friendly PDRC materials.

## Data Availability

The original contributions presented in this study are included in the article/Supplementary Materials. Further inquiries can be directed to the corresponding author.

## Supplementary Materials

The following supporting information can be downloaded at: https://www.mdpi.com/article/10.3390/polym18131580/s1. Figure S1: Fiber diameter distributions of the CaCO3/porous PLA nanofibers. Figure S2: SEM image of the PLA, CaCO3/PLA and porous PLA nanofibers. Figure S3: Fiber diameter distributions of the PLA, CaCO3/PLA and porous PLA nanofibers. Figure S4: (a) SEM and (b–e) mapping images for CaCO3/porous PLA nanofibers. Figure S5: TGA-DSC curves of CaCO3/porous PLA nanofibers. Figure S6: (a) Breathability test and (b) WVTR curves of PLA, CaCO3/PLA and Porous PLA nanofibers. Figure S7: Indoor and outdoor thermal images of CaCO3/porous PLA after 0 and 70 cycles. Figure S8: (a) Nighttime outdoor cooling performance and (b) temperature reduction achieved during outdoor testing of the various nanofibers. Figure S9: The residual mass of CaCO3/porous PLA nanofibers during burial. Table S1: Properties Comparison of Various Biodegradable Materials [23,34,54,55,56,57,58,59,60,61,62].
