# Supplementary material for: Electrospinning CaCO3/Porous PLA Nanofibers for Daytime Radiative Cooling
Source: Polymers (Basel). 2026 Jun 25;18(13):1580. doi: 10.3390/polym18131580 (PMC13363991; doi:10.3390/polym18131580)
Supplement: Supplementary file 1 [file polymers-18-01580-s001.zip › Revised Supplementary (Clean version).pdf]

Supplementary Discussion

Electrospinning of $CaCO_3$/Porous PLA Nanofibers for Daytime Radiative Cooling

Yangyang Sun[a,b], Changnai Yang[a], Mengge Li[a,b], Xiaomin Zeng[a], Dengkun Su[a], Shiyi Pan[a], Yu Zhang[b], Qiong Jiang[b], Shizhe Lin[a,*]

[a]Guangxi Key Laboratory of Optical and Electronic Materials and Devices, College of Materials Science and Engineering, Guilin University of Technology, Guilin, 541004, P. R. China

[b]Medical College of Guangxi University, Guangxi University, Nanning, 530004, P. R. China

*Corresponding author, e-mail: shizhelin@glut.edu.cn

Supplementary Calculations

For radiative cooling materials, the cooling power $P_{\text{cooling}}(T)$ under steady-state conditions, is defined as the energy balance between the emitted thermal radiation of the cooler and the absorbed heat fluxes from atmospheric radiation, solar irradiation, and non-radiative heat transfer:

$$P_{cooling}(T) = P_{rad}(T) - P_{atm}(T_{amb}) - P_{solar} - P_{(cond + conv)} \quad (1)$$

Where $P_{rad}(T)$ is the outward thermal radiation power emitted by the cooler, $P_{atm}(T_{amb})$ is the absorbed atmospheric thermal radiation, P_{solar} is the absorbed solar power, and $P_{(cond + conv)}$ represents non-radiative heat exchange, including conduction and convection.

The emitted thermal radiation power from the sample surface is calculated by integrating the spectral emissivity weighted by blackbody spectral radiance:

$$P_{rad}(T) = \int d\Omega \cos\theta \int_0^\infty d\lambda \, I_{BB}(T,\lambda)\varepsilon(\lambda,\theta) \quad (2)$$

Where θ is the polar angle relative to the surface normal, Ω is the upper-hemisphere solid angle, and $\varepsilon(\lambda, \theta)$ is the wavelength- and angle-dependent emissivity of the material.

The blackbody spectral radiance I_{BB} is given by Planck's law:

$$I_{BB}(T,\lambda) = \frac{2hc^2}{\lambda^5}\frac{1}{e^{hc/(\lambda k_B T)} - 1} \quad (3)$$

Where λ, c, h, and k_{B} denote wavelength, the speed of light ($3\text{x}10^{8}$ m s^{-1}), Planck's constant ($1.055\text{x}10^{-34}$ J s), and Boltzmann's constant ($1.381\text{x}10^{-23}$ J K^{-1}), respectively.

The absorbed atmospheric thermal radiation power is expressed as:

$$P_{atm}(T_{amb}) = \int d\Omega \cos\theta \int_0^\infty d\lambda I_{BB}(T_{amb},\lambda)\varepsilon(\lambda,\theta)\varepsilon_{atm}(\lambda,\theta) \quad (4)$$

Where T_{amb} is the ambient temperature and $\varepsilon_{\text{atm}}(\lambda, \theta)$ is the atmospheric emissivity.

The absorbed solar power is written as:

$$P_{solar} = \int_0^\infty d\lambda \, \varepsilon(\lambda,\theta_{solar}) I_{AM1.5}(\lambda) \quad (5)$$

Where $I_{\text{AM1.5}}(\lambda)$ is the AM1.5 standard solar spectral irradiance, and θ_{solar} is the solar incidence angle.

The non-radiative heat gains due to conduction and convection between the cooler and the surroundings are:

$$P_{cond + conv}(T, T_{amb}) = Ah_c(T_{amb} - T) \quad (6)$$

Where h_c is the non-radiative heat transfer coefficient. Considering heat exchange caused by conduction and convection between the cooling device, ambient air, and external surfaces, h_c is typically in the range of 0–12 W m^{-2} K^{-1}

The average solar reflectivity over 0.3–2.5 μm is defined as:

$$\overline{R}_{solar} = \frac{\int_{0.3\,\mu m}^{2.5\,\mu m} I_{AM1.5}(\lambda)\, R(\lambda)\, d\lambda}{\int_{0.3\,\mu m}^{2.5\,\mu m} I_{AM1.5}(\lambda)\, d\lambda} \tag{7}$$

The average emissivity within the atmospheric transparent window 8–13 μm is:

$$\bar{\varepsilon}_{8-13\mu m} = \frac{\int_{8\,\mu m}^{13\,\mu m} I_{BB}(T,\lambda)\, \varepsilon(T,\lambda)\, d\lambda}{\int_{8\,\mu m}^{13\,\mu m} I_{BB}(T,\lambda)\, d\lambda} \tag{8}$$

Where R is the reflectivity of the sample in the solar spectrum (0.3–2.5 μm), and $\varepsilon(T,\lambda)$ is the spectral thermal emissivity of the radiative cooler.

According to Kirchhoff's law, emissivity is calculated as:

$$\varepsilon = 1 - R - T \tag{9}$$

Where R and T are the infrared reflectivity and transmissivity of the material, respectively.

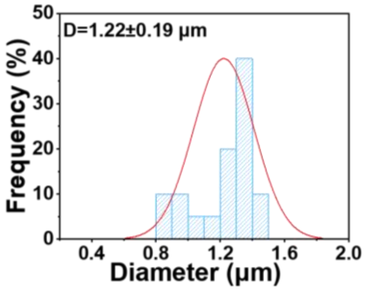

Figure S1. Fiber diameter distributions of the $CaCO_3$/porous PLA nanofibers.

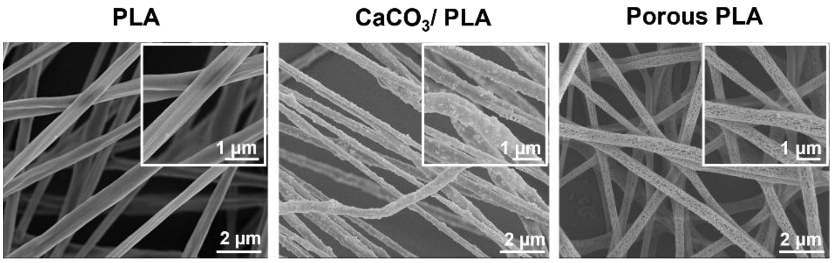

Figure S2. SEM image of the PLA, $CaCO_3$/ PLA and porous PLA nanofibers.

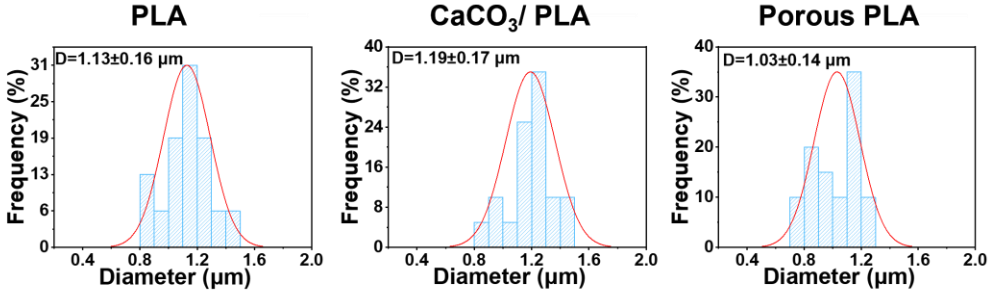

Figure S3. Fiber diameter distributions of the PLA, $CaCO_3$/ PLA and porous PLA nanofibers.

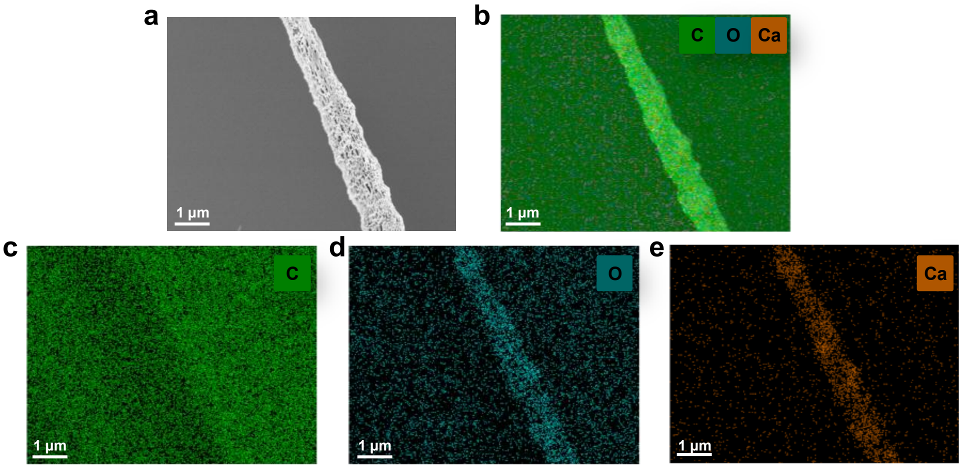

Figure S4. (a) SEM and (b-e) mapping images for $CaCO_3$/porous PLA nanofibers.

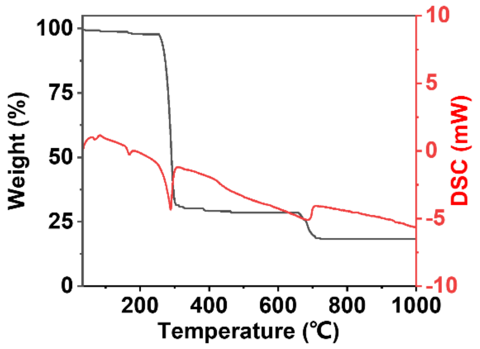

Figure S5. TGA-DSC curves of $CaCO_3$/porous PLA nanofibers.

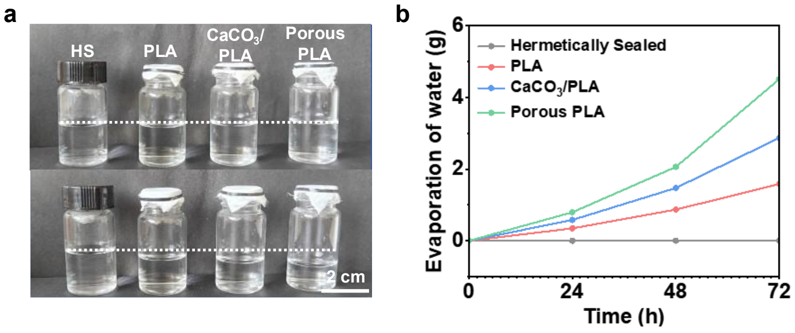

Figure S6 (a) Breathability test and (b) WVTR curves of PLA, $CaCO_3$/PLA and Porous PLA nanofibers.

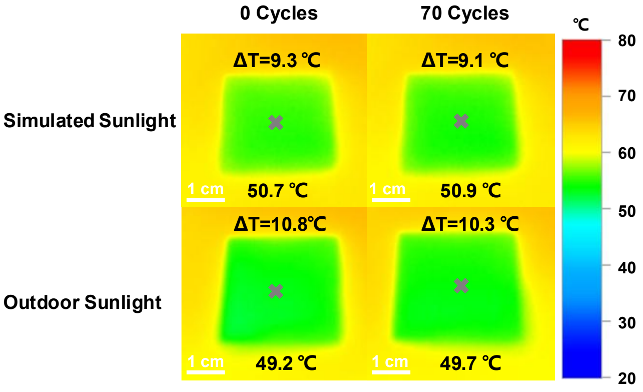

Figure S7. Indoor and outdoor thermal images of $CaCO_3$/porous PLA after 0 and 70 cycles.

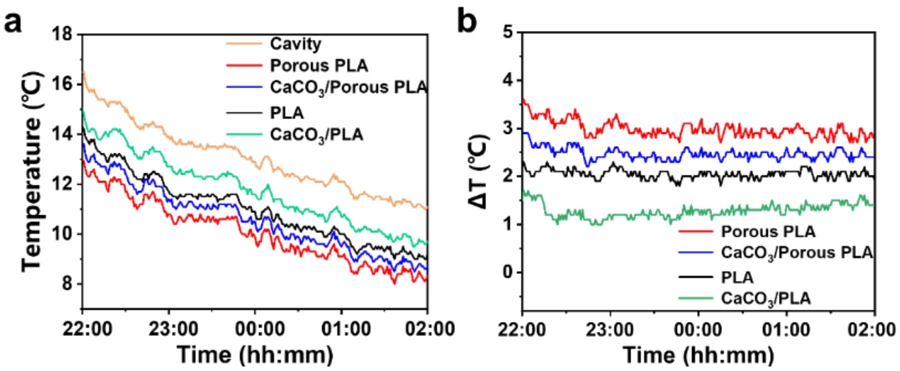

Figure S8. (a) Nighttime outdoor cooling performance and (b) temperature reduction achieved during outdoor testing of the various nanofibers.

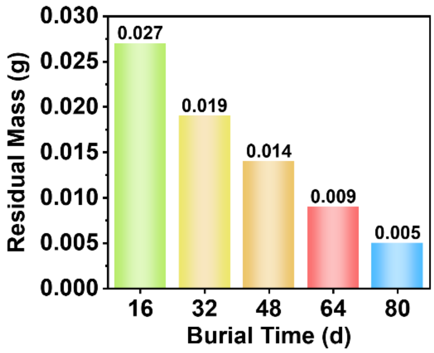

Figure S9. The residual mass of $CaCO_3$/porous PLA nanofibers during burial.

Table S1. Properties Comparison of Various Biodegradable Materials.

Sample	Solar Reflectivity (%)	Emissivity (%)	Temperature Reduction (℃)	Cooling Power (W m^{-2})	Mechanical Properties	Ref.
PLA/ZrO_2	77	94.31	9.14	———	9.0 kPa (compressive stress)	[1]
Cellulose Fibers	94	90	5	———	———	[2]
PLA Aerogel	92	90.5	3.7	———	0.15 MPa (compressive stress)	[3]
SiO_2/PHBV	95	89	12.8 (under human skin)	91.75	151% (elongation at break)	[4]
PLA	94.2	91.3	5.2	50.4	8.7 Mpa (tensile strength)	[5]
SF/PLA	96.1	95.4	6	———	———	[6]
Pu/D-$CaCO_3$	95	95	13	116.85	———	[7]
CNC/PVP	82.1	91.6	16.1	———	———	[8]
BCF (Bacterial Cellulose-basedcomposite Film)	———	94.6	7.15	———	———	[9]
KGM/PLA	91.06	91.4	10	———	———	[10]
CS-MA/PA	89.3	90.4	4.3-4.6	———	———	[11]
**$CaCO_3$/Porous PLA**	**92.3**	**91.6**	**10.3**	**96.1**	**1.66 MPa (compressive stress)**	**This work**

[1] Zhang S, Zhang Q, Huang X, et al. Robust structure and superhydrophobic PLA/ZrO_2 fiber aerogel for daytime radiative cooling[J]. *Adv. Ind. Eng. Polym. Res.*, **2025**, doi: 10.1016/j.aiepr.2025.10.001.

[2] Sun H, Tang F, Bi Y, et al. Hierarchically porous cellulose membrane via self-assembly engineering for ultra high-power thermoelectrical generation in natural convection[J]. *Adv. Funct. Mater.*, **2023**, 33(52): 2307960.

[3] Jia H, Mu M, Hou Y, et al. Template-thermally induced phase separation-assisted microporous regulation in poly (lactic acid) aerogel for sustainable radiative cooling[J]. *Biomacromolecules*, **2025**, 26(2): 1184-1194.

[4] Zhu Z J, Li Z, Wu X, et al. High-performance radiative cooling using a SiO_2/PHBV fiber membrane with a micronano-multistage structure[J]. *ACS Appl. Mater. Interfaces*, **2025**, 17(23): 34625-34636.

[5] Fan M, Hou Y, Jia H, et al. Sustainable Radiative Cooling of Microstructure Modulated Flexible poly (lactic Acid) Films[J]. *Langmuir*, **2025**, 41(31): 20768-20777.

[6] Xu P, Xiang B, Zhong W, et al. Biodegradable, scalable and flexible fiber membrane for green passive radiative cooling[J]. *Sol. Energy Mater. Sol. Cells*, **2023**, 253: 112209.

[7] Lee J, Kim D K, Kwon D, et al. Turning discarded oyster shells into sustainable passive radiative cooling films[J]. *Polymers*, **2025**, 17(3): 275.

[8] Li Z, Wang H, Yan X, et al. Versatile and Recyclable Iridescent Cellulose Nanocrystal Composite for Passive Daytime Radiative Cooling[J]. *ACS Appl. Mater. Interfaces*, **2026**, 18(11): 16980-16991.

[9] Ding Y C, Tang G W, Zhao H Y, et al. Scalable, flexible, and UV-resistant bacterial cellulose composite film for daytime radiative cooling[J]. *ACS Appl. Mater. Interfaces*, **2025**, 17(4): 6857-6866.

[10] Wang Y, Huang Z, Dai O, et al. Bionic porous konjac glucomannan/polylactic acid-based composite film: Synergistic functional design for antibacterial preservation and passive radiative cooling[J]. *Food Chem.*, **2025**: 147007.

[11] Cai W, Lin B, Qi L, et al. Bio-based and fireproof radiative cooling aerogel film: Achieving higher sustainability and safety[J]. *Chem. Eng. J.*, **2024**, 488: 150784.
